# A resource for functional investigation of miRNAs in rice responses to viral infection

**DOI:** 10.1111/pbi.14455

**Published:** 2024-08-24

**Authors:** Baogang Zhang, Xiong Zhang, Wenji Li, Dezhuo Pan, Baining Ma, Xinhui Duan, Chaoyi Dong, Lu Wang, Mingfu Zhao, Shanshan Zhao, Shuai Zhang, Jianguo Wu

**Affiliations:** ^1^ State Key Laboratory for Ecological Pest Control of Fujian and Taiwan Crops, College of Plant Protection Fujian Agriculture and Forestry University No.15 Shangshangdian Road, Cangshan District Fuzhou China; ^2^ Rice Research Institute Fujian Academy of Agricultural Sciences No. 247, Wusi Road, Gulou District Fuzhou China

**Keywords:** resource library, rice ragged stunt virus, rice grassy stunt virus, small RNA, viral resistance

Dear Editor,

In rice agriculture, the rice grassy stunt virus (RGSV) and rice ragged stunt virus (RRSV) present significant biosafety challenges. RGSV, a *Bunyaviridae* family virus with single‐stranded RNA and RRSV, a double‐stranded RNA virus from the Reoviridae family, are mainly transmitted by the brown planthopper, posing a co‐infection risk that can cause rice yellowing syndrome. This syndrome can severely hinder plant growth and, in extreme cases, lead to a complete yield loss. RNA interference (RNAi), featuring small RNA molecules such as microRNAs (miRNAs), emerges as a key antiviral defence, modulating gene expression by degrading or inhibiting translation of mRNA. This mechanism is integral to various plant biological processes, including growth, development and stress response. MiR168 and miR528, specifically, have been identified as vital in augmenting rice's resistance to viral attacks (Wu *et al*., 2015, 2017). Employing short tandem target mimic (STTM) technology has not only attenuated the function of certain miRNAs in rice but also underscored their role in essential agronomic traits (Zhang *et al*., 2017). Extending this approach, studies in maize and tomato demonstrate miRNAs' influence within hormonal signalling and secondary metabolism (Peng *et al*., 2018). While the regulatory networks of miRNAs in rice are well‐documented, the exploration of species‐specific miRNAs reactive to viral infections is relatively nascent.

To advance the understanding of species‐specific miRNAs in viral defence, this study established a transgenic rice library covering miRNA overexpression, suppression and gene knockout mutants mediated by CRISPR‐Cas9. Small RNA sequencing on rice stem base tissues infected with RGSV or RRSV identified 23 miRNAs with at least a two‐fold expression change relative to uninfected controls (Table S1). Heatmap analysis highlighted differential miRNA response patterns to RGSV and RRSV infections (Figure 1a). Validation by qRT‐PCR corroborated the altered expression of six *miRNAs* (*MIR440*, *MIR535*, *MIR1846d*, *MIR1863a*, *MIR1874‐3p* and *MIR1881*), consistent with sequencing results. Notably, miR440, miR1846d, miR1863a and miR1874‐3p were predominantly downregulated by RGSV, whereas miR535 and miR1881 were suppressed by both viruses (Figure 1b,c). To elucidate miRNA roles in viral pathogenesis, we engineered three vector types for precise miRNA regulation. The overexpression vector was constructed by cloning the miRNA precursor with ~100 bp flanking sequences downstream of the Actin1 promoter (Figure S1a). For knockdown, the STTM technology was employed; we fused the miRNA complementary sequence plus three nucleotides to a 48 nt linker, and inserted this downstream of the Actin1 promoter to create the STTM vector (Figure S1b). Additionally, we designed a CRISPR‐Cas9 editing vector by targeting two sites on the miRNA precursor sequence (Figure S1c). We successfully introduced 68 vectors into the ZH11 rice variety using Agrobacterium‐mediated transformation, achieving 20 overexpression, 22 STTM‐mediated knockdown and 20 CRISPR‐Cas9‐mediated knockout events. Each event type was confirmed in at least two independent transgenic lines, fulfilling the expected experimental criteria (Figures S2–S4; Tables S2 and S3). The phenotypic profiles of select transgenic lines at the booting stage are documented in Figure S5. In T2 generation homozygous lines, we performed inoculation trials with RGSV and RRSV to determine the miRNAs' roles in rice‐virus interactions. Post‐RGSV inoculation, the overexpression line *OX535* demonstrated a significant susceptibility rate of 76.9%, while the *MIM535* suppression line and the *mir535* knockout line showed enhanced resistance (Table S4). *MIR535* is ubiquitously expressed in rice tissues, including rhizomes, stem base, glumes and germinated seeds (Figure S6). Furthermore, RGSV infection was found to downregulate *MIR535* promoter activity, reducing *MIR535* transcript levels (Figures 1b,d and S7). Pathogenic assessments and virus titre analyses revealed that the *OX535* line accumulated greater viral loads and exhibited more severe symptoms, such as pronounced dwarfing and increased tillering. In contrast, the *MIM535* suppression line and the *mir535* knockout line presented with lower virus titre and milder symptoms (Figure 1e–g). Under non‐stress conditions, *OX535* displayed dwarfism, multiple tillering, smaller spikes and reduced spikelet branching, but grains were longer and wider. No significant variances were observed in the *MIM535* and *mir535* lines for key agronomic traits, including plant height, tiller number, spike size, grain dimensions and 100‐grain weight, compared to the ZH11 variety (Figures 1h–n and S8). The enhanced viral resistance of these lines identifies miR535 as a promising target for RGSV resistance breeding.

**Figure 1:**
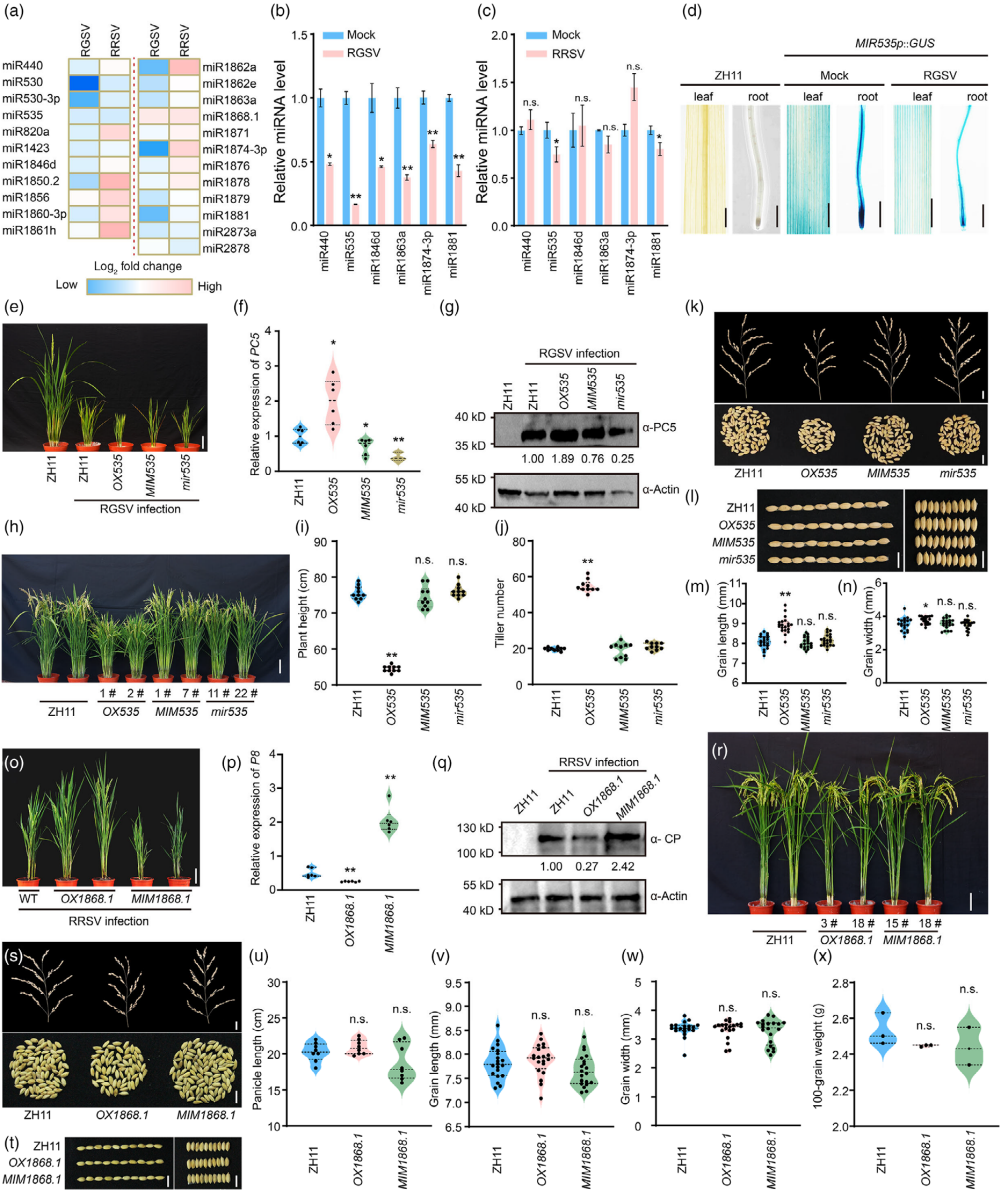
Exploring miRNA Function in Rice‐Virus Interactions. (a) Heatmap depicts miRNA log‐fold changes in rice infected with RGSV and RRSV versus control ZH11. (b, c) qRT‐PCR confirms miRNA levels post‐RGSV (b) and RRSV (c) infection. (d) GUS staining demonstrates the impact of RGSV on *MIR535* promoter activity in rice. (e) Disease manifestations in transgenic lines post‐RGSV infection; scale bar, 10 cm. (f, g) qRT‐PCR (f) and Western blot (g) analyses quantify the RGSV coat protein PC5 in infected transgenic rice. (h) Morphology of transgenic lines at the tillering stage; scale bar, 10 cm. (i, j) Measured plant heights (i) and tiller numbers (j) in the transgenic lines. (k) Upper panel: number of primary branches per main panicle (scale bar, 2 cm). Lower panel: grain count per main panicle (scale bar, 1 cm). (l) Grain size of transgenic lines with the left panel showing 10‐grain length and the right panel showing 10‐grain width. (m, n) Statistical evaluations of grain length (m) and width (n). (o) Disease symptoms in transgenic lines after RRSV infection; scale bar, 10 cm. (p, q) qRT‐PCR (p) and Western blot (q) measure RRSV coat protein in infected transgenic plants. (r) Morphology of *OX1868.1* and *MIM1868.1* transgenic rice. Scale bar, 10 cm. (s) Upper panel: primary branch count per main panicle (scale bar, 2 cm). Lower panel: grains per main panicle (scale bar, 1 cm). (t) 10‐grain dimensions, with the left panel for length and the right for width; scale bar, 1 cm. (u–x) Statistical analyses of panicle length (u), grain length (v), width (w) and 100‐grain weight (x), with asterisks denoting significant differences (*t*‐test, *P ≤ 0.05, **P ≤ 0.01).

Rice ragged stunt virus resistance screening indicated that the *OX1868.1* line exhibited enhanced viral resistance, while the *MIM1868.1* line was more susceptible to the virus. Multiple lines of evidence indicate that miR1868.1 can suppress RRSV accumulation. This includes quantifying viral transcript levels using RT‐qPCR, and detecting the expression levels of viral coat proteins through Western blot analysis (Figure 1o–q). Agronomic trait assessments showed no significant differences in panicle length, grain size and 100‐grain weight between *OX1868.1*, *MIM1868.1* and the ZH11 control, although *OX1868.1* exhibited reduced tillering and decreased plant height (Figures 1r–x and S9). Consequently, miR1868.1 demonstrates antiviral activity against RRSV and represents a valuable target for rice antiviral breeding programs.

The research has successfully established a miRNA resource library with modalities for overexpression, mimicry (MIM) and knockout of miRNAs. This library facilitates the modelling of gene loss‐of‐function and the resulting phenotypic manifestations. Of the 23 miRNAs curated, 16 exist as single copies in rice, potentially increasing the effectiveness of CRISPR‐Cas9 knockouts. Remarkably, 19 miRNAs are so far exclusive to rice, indicating a library enriched with species‐specific sequences. Beyond the previously mentioned miR535 and miR1868.1, this work extends to other miRNAs involved in the viral resistance of rice. The genetic constructs are also valuable for investigating rice growth, development and response to biotic and abiotic stress. Overall, this initiative has created a transgenic rice miRNA library, spotlighting 23 miRNAs pivotal to viral infection responses.

## Conflict of interest

The authors have declared no conflict of interest.

## Author contributions

B.Z., S.‐S.Z. and J.W. designed the experiments. B.Z., X.Z., W.L., D.P., B.M., X.D., C.D., L.W. and M.Z. conducted the experiments and analysed the data. B.Z. and S.Z. wrote the paper with the input of all other authors.

## Supporting information


**Figure S1** Schematic representation of constructs for miRNA overexpression, knockdown, and knockout in plants.
**Figure S2** Relative miRNA levels in various overexpression transgenic rice lines.
**Figure S3** Relative miRNA levels in various miRNA mimic rice lines.
**Figure S4** Northern blot analysis of miR535 and miR1868.1 expression in rice.
**Figure S5** Morphological comparison of ZH11 and miRNA transgenic rice lines.
**Figure S6** Tissue‐specific expression of *MIR535* in transgenic rice.
**Figure S7** Suppression of *MIR535* expression by RGSV.
**Figure S8** Agronomic trait assessment in rice lines with modified miRNA expression.
**Figure S9** Effects of *OX1868.1* and *MIM1868.1* on tiller number and plant height in rice.

**Table S1** Reads of screened miRNAs in small RNA‐seq data.

**Table S2** Detailed information of screened miRNAs.

**Table S3** CRISPR‐Cas9‐mediated genome edition in miRNA precursor region.

**Table S4** Disease incidence of RGSV‐ or RRSV‐inoculated rice plants.

**Table S5** Oligonucleotides used in this study.

## Data Availability

The data that supports the findings of this study are available in the supplementary material of this article.
