# Supplementary material for: A resource for functional investigation of miRNAs in rice responses to viral infection
Source: Plant Biotechnol J. 2024 Aug 24;22(12):3380–2. doi: 10.1111/pbi.14455 (PMC11606404; doi:10.1111/pbi.14455)
Supplement: Supplementary file 1 — Figure S1 Schematic representation of constructs for miRNA overexpression, knockdown, and knockout in plants. Figure S2 Relative miRNA levels in various overexpression transgenic rice lines. Figure S3 Relative miRNA levels in various miRNA mimic rice lines. Figure S4 Northern blot analysis of miR535 and miR1868.1 expression in rice. Figure S5 Morphological comparison of ZH11 and miRNA transgenic rice lines. Figure S6 Tissue‐specific expression of MIR535 in transgenic rice. Figure S7 Suppression of MIR535 expression by RGSV. Figure S8 Agronomic trait assessment in rice lines with modified miRNA expression. Figure S9 Effects of OX1868.1 and MIM1868.1 on tiller number and plant height in rice. [file PBI-22-3380-s006.docx]

**
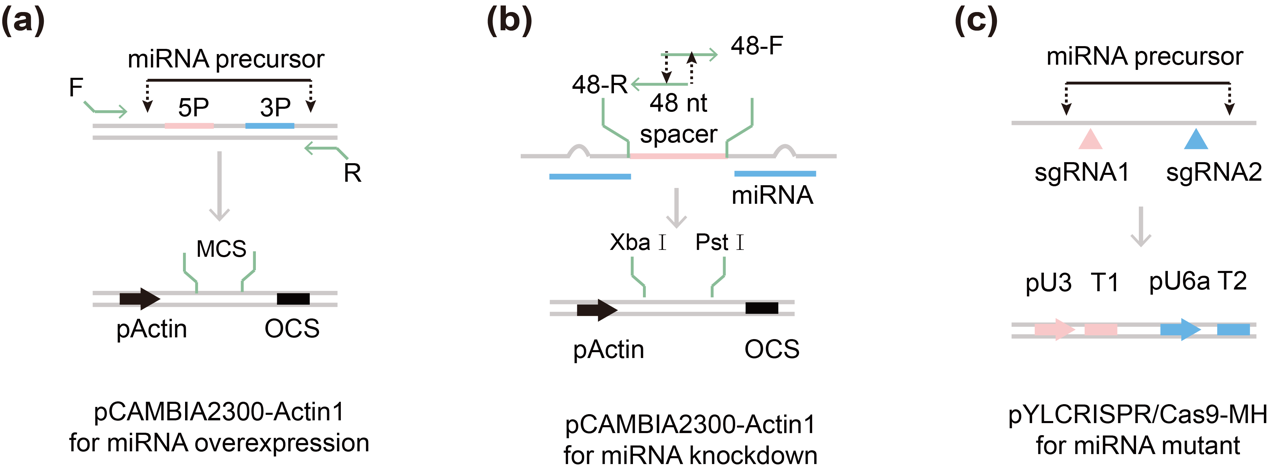
**

**Figure S1. Schematic Representation of Constructs for miRNA Overexpression, Knockdown, and Knockout in Plants.**

**(a)** Vector construction for miRNA overexpression, featuring sequences approximately 100 base pairs upstream and downstream of the miRNA precursor cloned into the pCAMBIA2300-Actin1 vector at the multi-cloning site (MCS), flanked by the pActin1 promoter and the OCS terminator. **(b)** Strategy for miRNA knockdown involves fusing the antisense complementary sequence of the miRNA with three additional nucleotides, using fusion PCR based on the STTM technology with a 48 nucleotide (nt) spacer. This fusion construct is then integrated downstream of the actin1 promoter to create the knockdown expression vector using *Xba* I and *Pst* I restriction sites. **(c)** CRISPR/Cas9-mediated miRNA knockout setup, illustrating the placement of sgRNA1 and sgRNA2 targeting the miRNA precursor in the pYLCRISPR/Cas9-MH vector, driven by the pU3 T1 and pU6a T2 promoters.


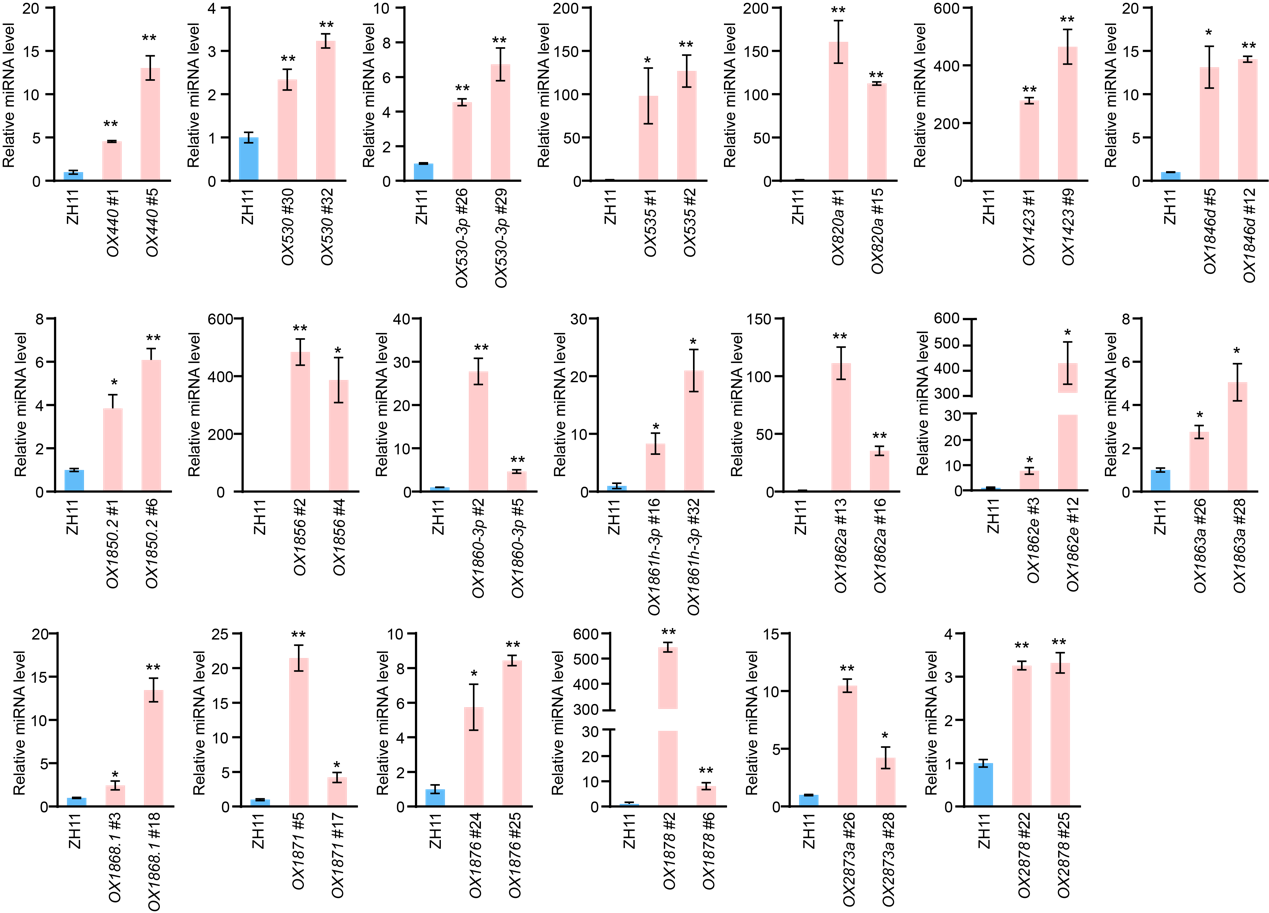


**Figure S2. Relative miRNA Levels in Various Overexpression Transgenic Rice Lines.**

The data show the relative miRNA levels in ZH11 rice and the corresponding overexpression lines. Transgenic strains of *OX1874-3p*, *OX1879*, and *OX1881* were not available in the resource, and no corresponding material was identified. Error bars representing standard deviation from three biological replicates (n=3). Statistical significance compared to ZH11 is denoted by asterisks: *, *P* ≤ 0.05, **, *P* ≤ 0.01 as determined by Student's *t*-test.


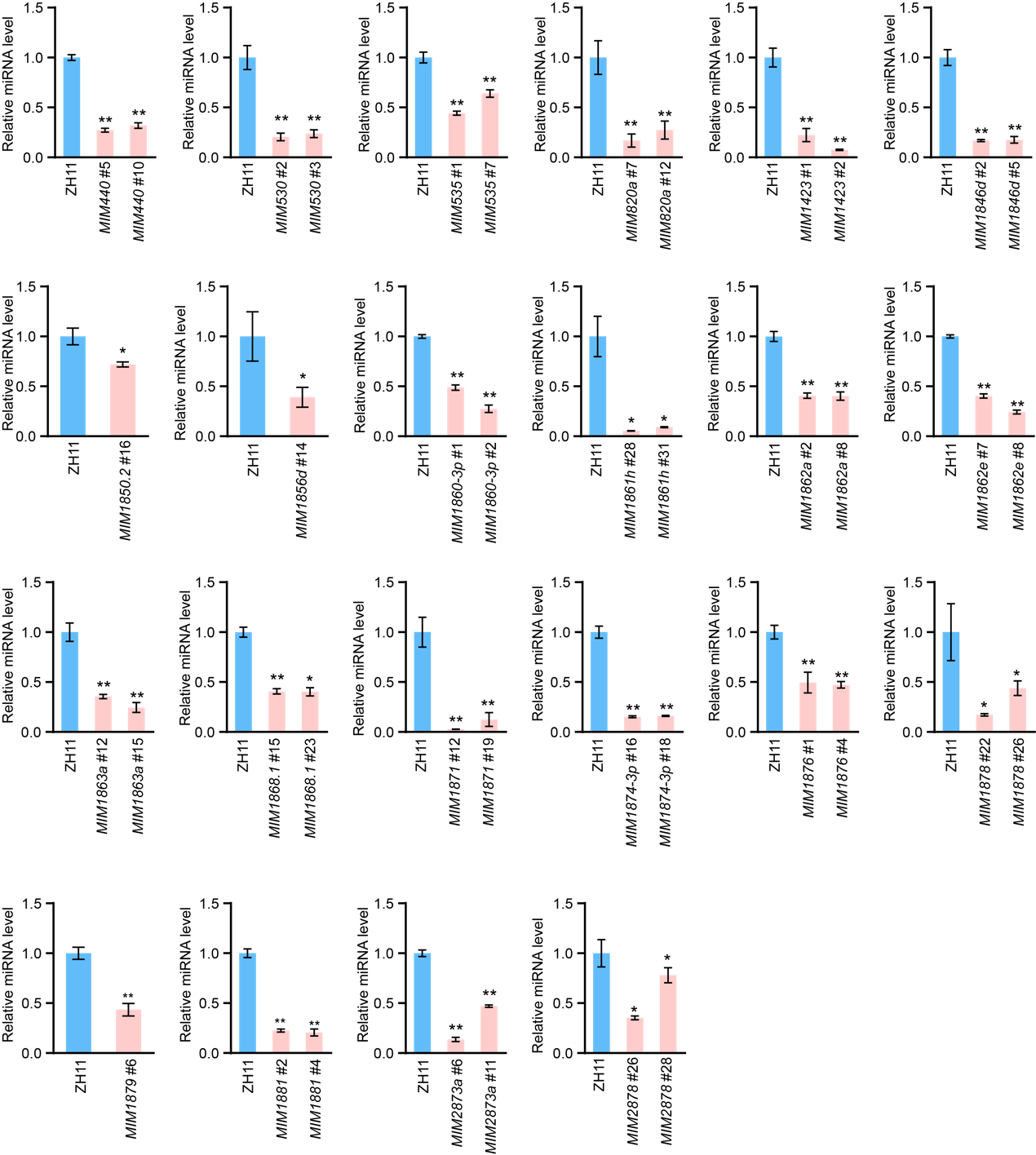


**Figure S3. Relative miRNA Levels in Various miRNA mimic Rice Lines.**

The relative miRNA levels were quantified using qRT-PCR and are displayed as fold changes relative to the wild-type ZH11. Transgenic strains of *MIM530-3p* were not available in the resource, and no corresponding material was identified. Error bars represent the standard deviation from triplicate samples (n=3). Statistical significance, determined by Student's *t*-test, is indicated by asterisks: ***, *P ≤ 0.05, ***, *P ≤ 0.01*.


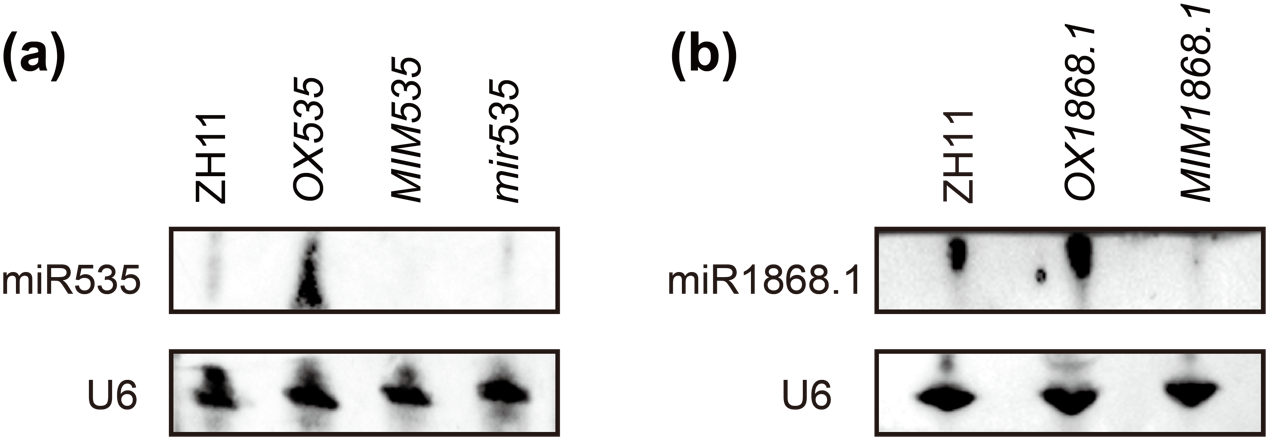


**Figure S4: Northern blot analysis of miR535 and miR1868.1 expression in rice.**

**(a)** Detection of miR535 levels in ZH11 control, OX535 overexpression, and MIM535 mimic lines. **(b)** Detection of miR1868.1 levels in ZH11 control, OX1868.1 overexpression, and MIM1868.1 mimic lines. U6 used as a loading control to normalize the expression levels of the microRNAs.


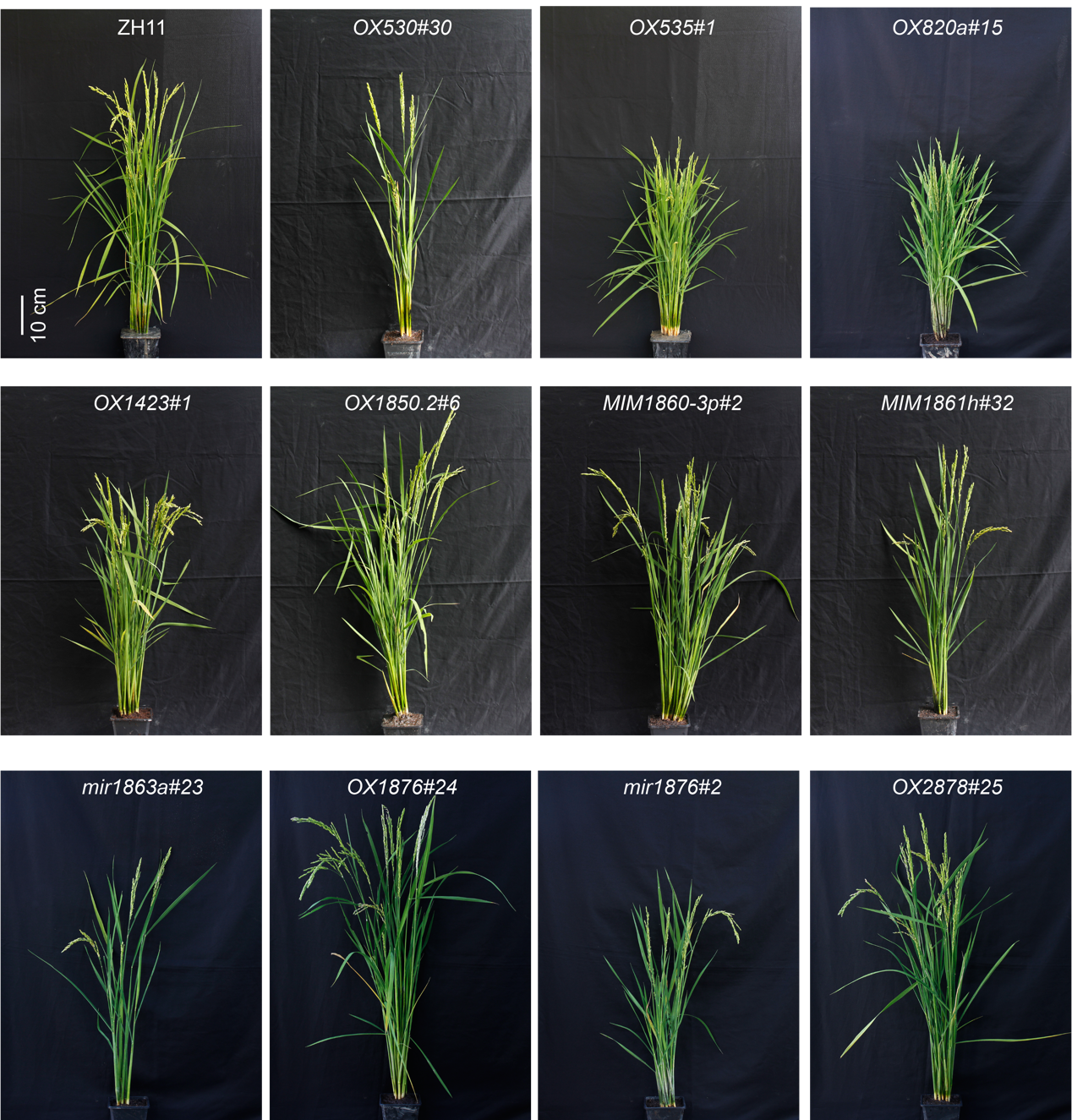


**Figure S5. Morphological Comparison of ZH11 and miRNA Transgenic Rice Lines.**

Photographs displaying the overall plant growth and architecture at the reproductive stage. The images showcase variations in plant development among the different genotypes. Scale bar, 10 cm.


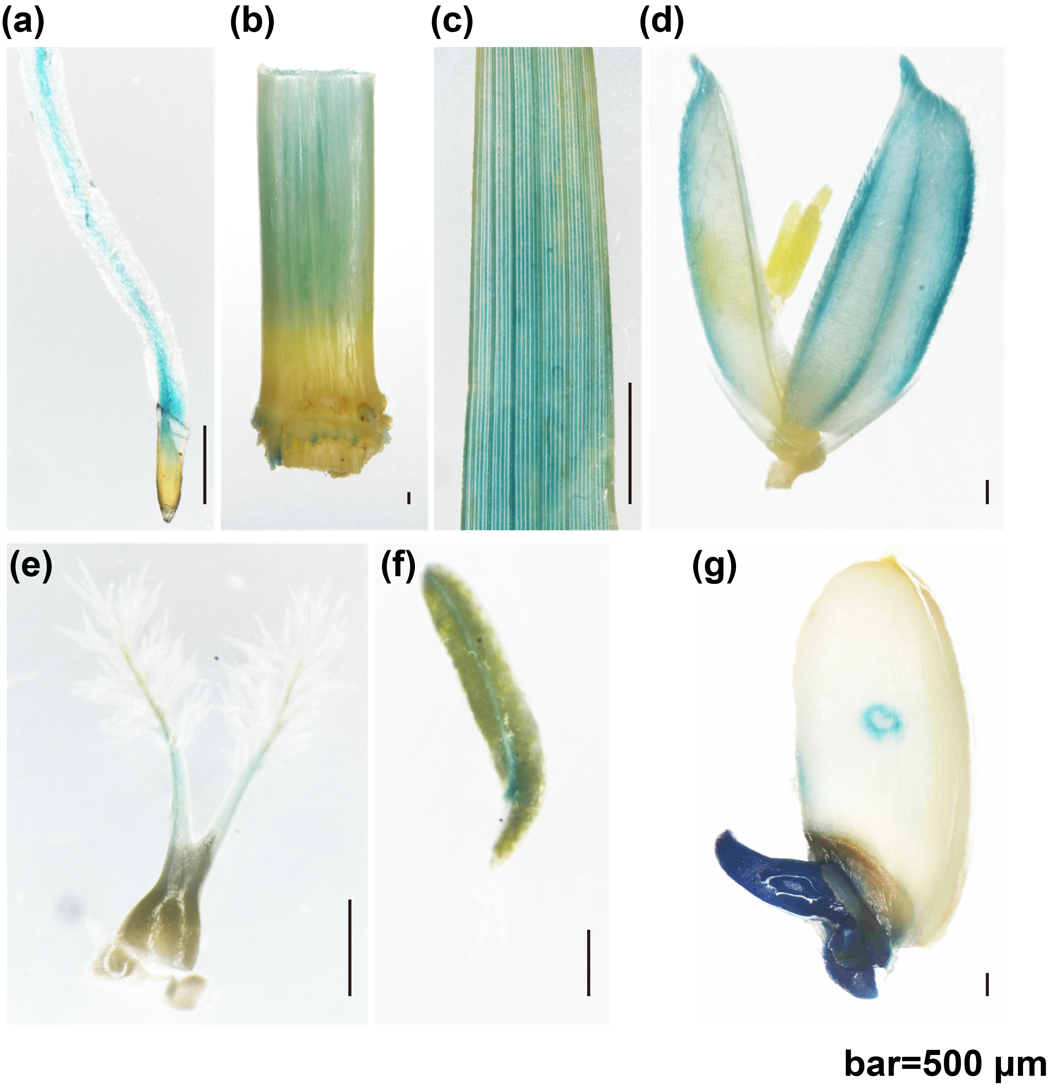


**Figure S6. Tissue-Specific Expression of *MIR535* in Transgenic Rice.**

GUS staining demonstrates the expression of the *MIR535* promoter in various tissues of transgenic rice plants harboring the *MIR535*p::GUS construct. **(a)** Root tip, **(b)** Stem base, **(c)** Leaf blade, **(d)** Glumes, **(e)** Pistil, **(f)** Anther, and **(g)** Germinated seed. The blue staining indicates the activity of the *GUS* reporter gene, reflecting the expression pattern of *MIR535*. Scale bars, 500 μm.


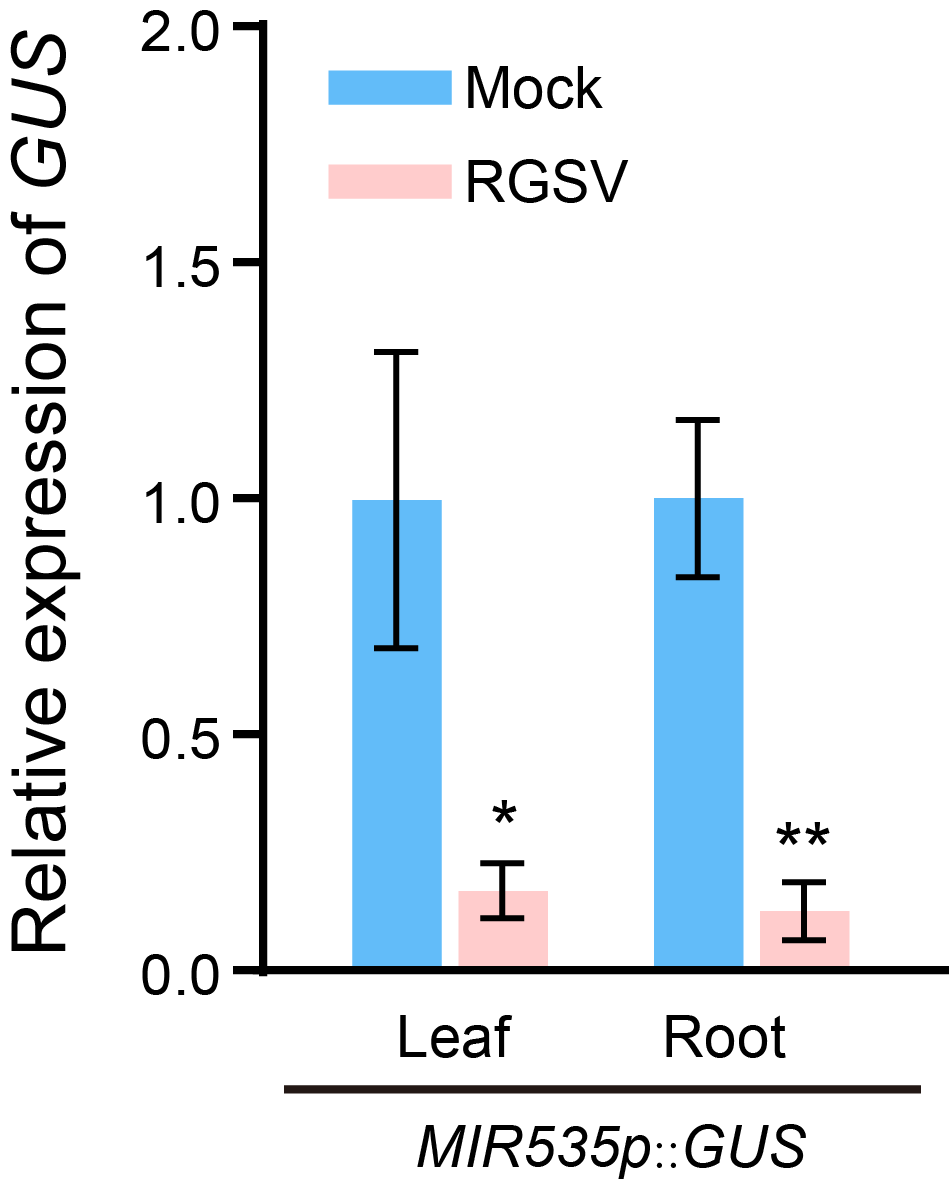


**Figure S7. Suppression of *MIR535* Expression by RGSV.**

Quantitative RT-PCR analysis of *GUS* expression in rice root and leaf tissues infected with RGSV (n=3). Error bars represent the standard deviation (SD). *P*-values were calculated using Student's *t*-test: *, *P* ≤ 0.05; **, *P* ≤ 0.01.


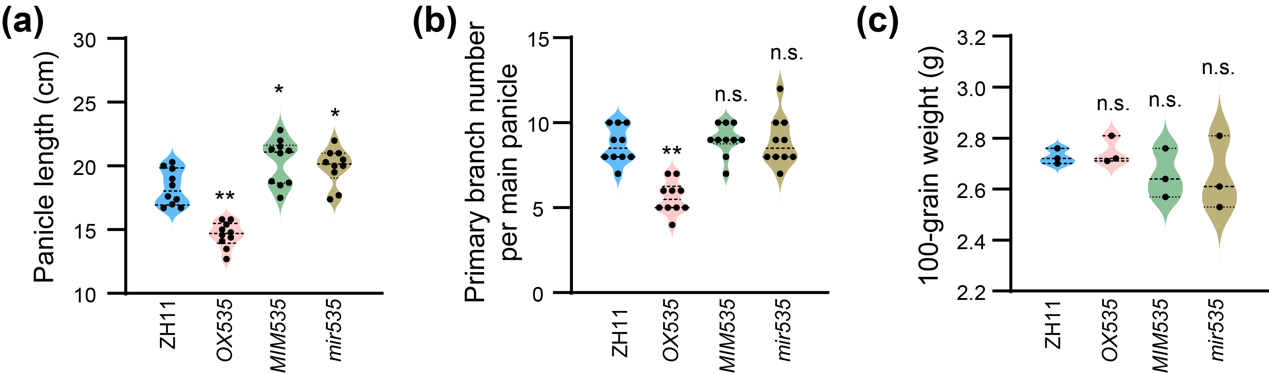


**Figure S8. Agronomic Trait Assessment in Rice Lines with Modified miRNA Expression**

**(a)** Violin plots showing panicle length measurements across different rice lines: ZH11, *OX535* overexpression line, *MIM535* mimic line, and *mir535* knockdown line. Each point represents an individual measurement, with the overall distribution indicated. **(b)** Primary branch number per main panicle comparison among the same rice lines, illustrating potential differences in branching due to miRNA expression modulation. **(c)** Hundred grain weight data for each line, providing insight into the effects of miRNA expression changes on grain weight. For all panels, asterisks indicate statistical significance determined by Student's *t*-test (*, *P* ≤ 0.05, **, *P* ≤ 0.01), and 'n.s.' denotes not significant. Error bars represent standard deviation.


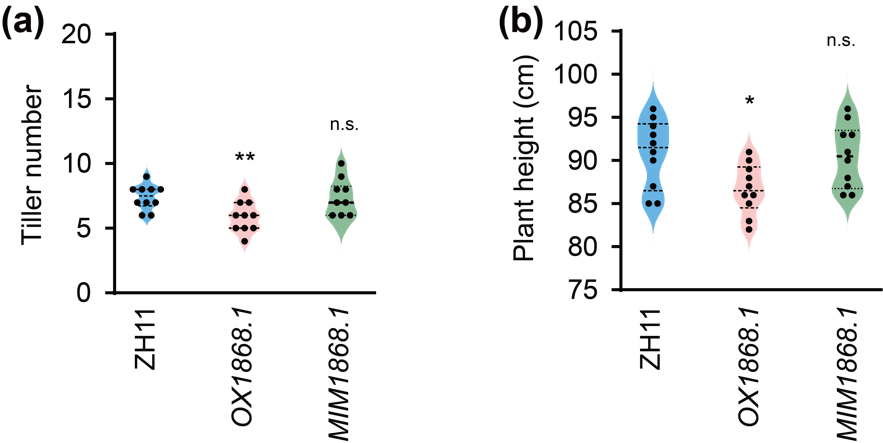


**Figure S9: Effects of *OX1868.1* and *MIM1868.1* on tiller number and plant height in rice.**

**(a)** Tiller number in rice lines ZH11 (control), *OX1868.1* (overexpression), and *MIM1868.1* (mimic). **(b)** Plant height measurements for the same rice lines. Asterisks indicate statistical significance determined by Student's *t*-test (*, *P* ≤ 0.05, **, *P* ≤ 0.01), and 'n.s.' denotes not significant.
